# Mechanistic Insight into the Relationship between N-Terminal Acetylation of α-Synuclein and Fibril Formation Rates by NMR and Fluorescence

**DOI:** 10.1371/journal.pone.0075018

**Published:** 2013-09-18

**Authors:** Lijuan Kang, Maria K. Janowska, Gina M. Moriarty, Jean Baum

**Affiliations:** 1 Department of Chemistry and Chemical Biology, Rutgers University, Piscataway, New Jersey, United States of America; 2 BioMaPS Institute for Quantitative Biology, Rutgers University, Piscataway, New Jersey, United States of America; Consejo Superior de Investigaciones Cientificas, Spain

## Abstract

Aggregation of α-synuclein (αSyn), the primary protein component in Lewy body inclusions of patients with Parkinson’s disease, arises when the normally soluble intrinsically disordered protein converts to amyloid fibrils. In this work, we provide a mechanistic view of the role of N-terminal acetylation on fibrillation by first establishing a quantitative relationship between monomer secondary structural propensity and fibril assembly kinetics, and secondly by demonstrating in the N-terminal acetylated form of the early onset A53T mutation, that N-terminal transient helices formed and/or inhibited by N-terminal acetylation modulate the fibril assembly rates. Using NMR chemical shifts and fluorescence experiments, we report that secondary structural propensity in residues 5–8, 14–31, and 50–57 are highly correlated to fibril growth rate. A four-way comparison of secondary structure propensity and fibril growth rates of N-terminally acetylated A53T and WT αSyn with non-acetylated A53T and WT αSyn present novel mechanistic insight into the role of N-terminal acetylation in amyloid fibril formation. We show that N-terminal acetylation inhibits the formation of the “fibrillation promoting” transient helix at residues 14–31 resulting from the A53T mutation in the non-acetylated variant and supports the formation of the “fibrillation inhibiting” transient helix in residues 1–12 thereby resulting in slower fibrillation rates relative to the previously studied non-acetylated A53T variant. Our results highlight the critical interplay of the region-specific transient secondary structure of the N-terminal region with fibrillation, and the inhibitory role of the N-terminal acetyl group in fibril formation.

## Introduction

Protein misfolding is the origin of a wide variety of human diseases including Alzheimer’s, Parkinson’s and Prion disease as well as amyloid-related type II diabetes [1,2,3,4]. These diseases are associated with proteins that convert from their normally soluble form to large aggregates including amyloid fibrils that accumulate in the brain or affected organs [5,6]. As life expectancy continues to increase, debilitating diseases like Parkinson’s are becoming increasingly common and effective strategies for fighting them are essential. The diagnostic hallmark of Parkinson’s disease (PD) is a deposit called a Lewy body that is primarily composed of the amyloid fibril form of α-synuclein (αSyn) [7,8]. The physiological function of αSyn is not fully understood, but it is involved in synaptic vesicle trafficking [9,10], regulation of the synaptic vesicle pool [11], and may act as a non-classical chaperone that promotes the assembly of a SNARE-complex in neuronal synapses [12,13].

**Figure 1:**
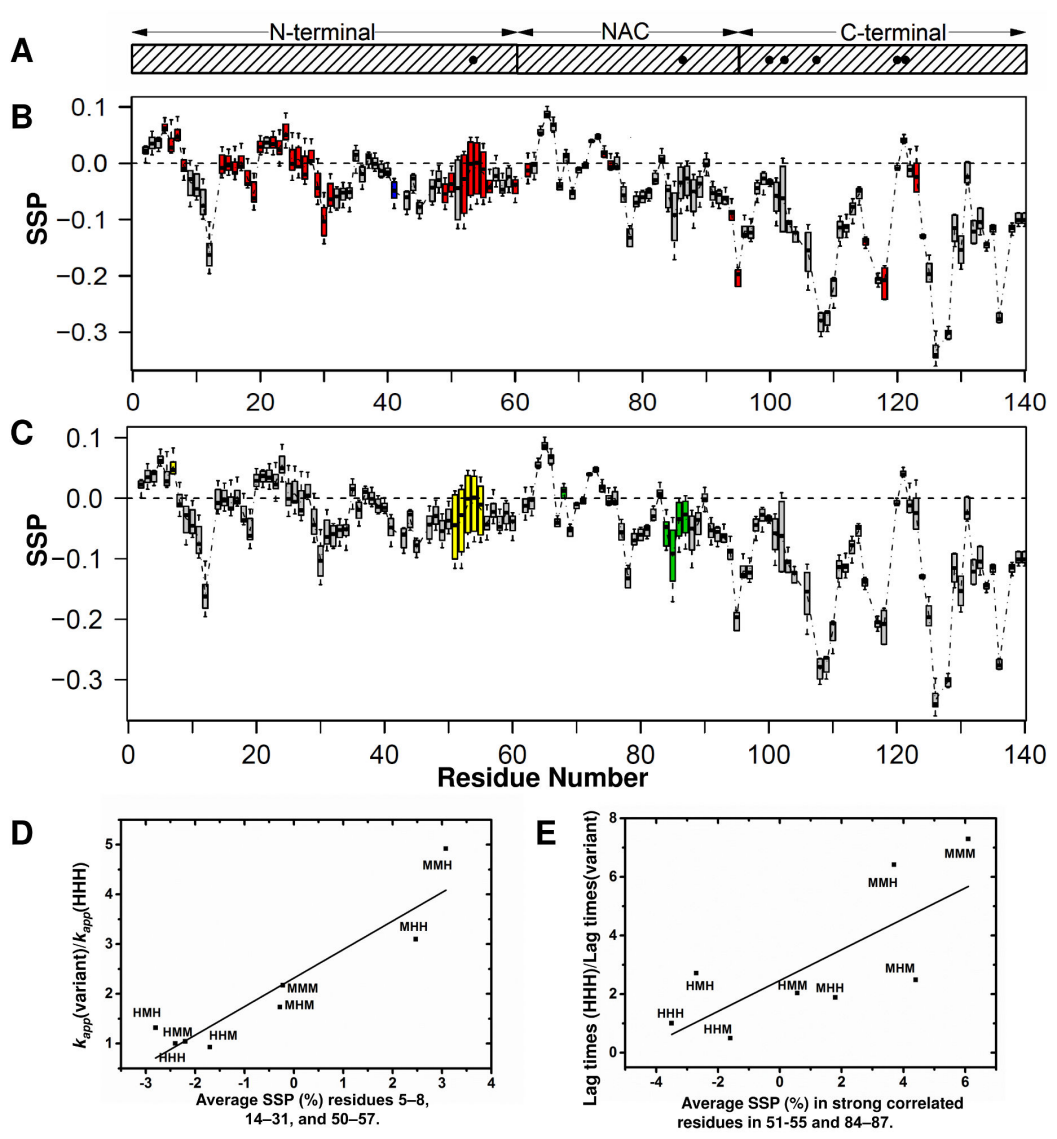
Quantitative correlation of SSP values to fibril kinetics in the non-acetylated human-mouse chimera αSyn set. A. The N-, NAC, and C-terminal regions are shown in the schematic representation of αSyn. The black dots represent seven possible substitutions between human and mouse WT αSyn. B. Boxplot diagram representing the dispersion of SSP values for each human-mouse chimera as a function of residue, where each boxplot is a five-number summary of the SSP distribution of the eight variants. Residues are shaded based on their correlation with the growth rate (k_app_), data from a previous study [39]. Residues with high positive correlation are shaded in red (r > 0.7), high negative correlation in blue (r < -0.7) and no correlation are shown as grey (-0.7 < r < 0.7). C. The representation is the same as in Figure 1B, however, the boxplots are shaded based on correlation to lag times. Residues with highest positive correlation are shaded in light red (r > 0.5), with highest negative correlation are shaded in light blue (r < -0.5) and no correlation with grey (-0.5 < r < 0.5). D. Correlation between the average SSP in regions that have a strong correlation with *k*_*app*_ determined by Figure 1B (residues 5–8, 14–31, and 50–57) and *k*_*app*_(variant)/*k*_*app*_(HHH) for all eight variants, of which details of naming and BMRB accession numbers are indicated in Table 1. The correlation coefficient is r = 0.93. The correlation function is Y = (2.32±0.2)+ (0.57±0.9)* X.E. Correlation between the average SSP in regions that have strong correlation with lag times determined by Figure 1C (residues 51–55 and 84–87) and lag times (HHH)/lag times (variant) for all eight variants. The correlation coefficient is r = 0.75. The correlation function is Y = (2.46±0.66) + (0.53±0.19)* X.

αSyn has been considered to be an intrinsically disordered protein with high net charge and low hydrophobic content [14]. It is a 140 amino acid protein consisting of three domains: 1) an N-terminal domain (residues 1–60) that supports regions of transient α-helical propensity; 2) a central hydrophobic region, known for historical reasons as the “non-Amyloid β component” (NAC) (residues 61–95) that is itself highly amyloidogenic and forms the core of the amyloid fibril [15,16]; and 3) a C-terminal region that is acidic and proline-rich (residues 96–140) [17] (Figure 1A). Recent reports have suggested that αSyn may exist as a tetramer when purified from red blood cells and neuronal cell lines [18], but subsequent comprehensive studies by an assemblage of groups, including our own, have shown that αSyn exists predominately as a disordered monomer [19,20,21,22].

Alterations to the monomer including mutations or modifications that affect the aggregation of αSyn, particularly fibril kinetics, have been a great focus of current PD research. While most cases of PD are sporadic, approximately 10% of the cases arise from familial mutations including A53T, A30P and E46K. A53T was the first familial mutation initially linked to PD, as A53T monomers are consumed into fibrils more rapidly than the wild type (WT) [23], suggesting the correlation of amyloid to disease. We therefore previously examined a set of human-to-mouse αSyn chimeras to probe the effect of the A53T mutation, as the N-terminus of mouse αSyn interestingly differs from human only in a substitution from A to T at position 53. By exploring systematic regional substitutions between mouse and human αSyn, we were able to demonstrate that the presence of the A53T mutation dominated accelerated fibril kinetics of the set. This acceleration was linked to the observed transient helical propensity around the site of mutation, suggesting that preformed transient secondary structure of the monomer plays a critical role in directing fibrillation.

In this report, we add to this mechanistic picture by investigating how the conformational effects in the monomer arising from N-terminal acetylation affect the fibril kinetics of the A53T mutant. N-terminal acetylation is an important co-translational modification event in eukaryotes, and a ubiquitous event upon the αSyn monomer in particular. It has been previously demonstrated that both soluble and insoluble fractions from brain tissues in patients suffering from dementia with Lewy bodies contain N-terminally acetylated αSyn (Ac-WT) [24], and it was recently shown that the mass of αSyn in the proposed tetrameric form of the protein purified from red blood cells and neuronal cell lines corresponds to the modification of the monomer by an acetyl group [18]. In response to detection of this modification, our group, along with others, has recently investigated the effects of N-terminal acetylation *in vitro* on αSyn conformation and fibril formation [20,21]. We have shown that an increase in transient helical secondary structure at the N-terminus (residues 1–12) corresponds to a roughly two-fold slowing of fibril growth rate (*k*_*app*_) relative to non-acetylated WT αSyn. This increased helical structure has been rationalized as the effect of N-capping and stabilization of the helix dipole by removal of the α-amino positive charge [20]. A mechanistic view of the role of N-terminal acetylation in fibrillation and the nature of the relationship between the transient helix at residues 1–12 and delayed fibril assembly is not yet understood, but a similar slowing of fibrillation by acetylation of the A53T mutant further suggests a region-dependent role of transient helix in directing fibril formation.

A relationship between the monomer species and fibril assembly kinetics has been suggested by many laboratories studying amyloidogenic proteins, however, at this stage, there are differing views as to the nature of the critical conformational requirements of the αSyn monomer for fibril initiation. Alongside ours and others’ view that inherent secondary structural propensities of the αSyn monomer are the critical directors of fibrillation, recent proposals for amyloidogenic IAPP and Aβ similarly point to involvement of helical intermediates in fibril formation, particularly during the lag phase. In these cases, stabilization of helical conformations in the monomer may similarly accelerate the formation of β-sheet rich aggregate structures [25,26,27,28,29,30,31,32]. Some other evidence supports an alternative view of αSyn fibrillation. There are proposals that the release of long-range interactions which exposes the hydrophobic NAC region, is the critical fibrillation initiator [33,34]. Some evidence indicates rather that physico-chemical properties such as the electrostatic charge and hydrophobicity of a disordered protein [35,36,37,38], may, irrespective of any transient structure observed in the monomeric form, determine the local fibrillation propensity within a given amino acid sequence. While the fibrillation kinetics of αSyn are likely dominated by some combination of factors, we demonstrate in this report that transient secondary structure of the monomer N-terminus is clearly a quantitatively correlated observable. If the controversy in the field about the relationship between the nature of the monomer ensemble and fibril formation can be resolved, at least in part by the type of suggestions we propose in this report, this will help to establish a mechanistic view of αSyn fibril assembly.

In this work, we extend our view of monomer transient secondary structure as a director of fibrillation by centering our NMR and fluorescence observations on the acetylated-A53T mutant and on both of its modifications from the WT: N-terminal acetylation and the A53T mutation. Using the human-to-mouse chimera set, which includes the A53T mutations, we provide a quantitative correlation of secondary structural propensity to *k*_*app*_ and to lag phase of fibrillation. The effect of acetylation upon fibrillation relative to the non-acetylated in both the WT and, as demonstrated in this report, A53T, strongly implies the inhibitory role of acetylation in the four-way comparison we present here. These studies have identified that transient helix in different regions within the N-terminus have the potential to be either fibrillation inhibiting or fibrillation promoting, and we show that alterations from the WT, either by N-terminal acetylation or by mutation, can influence the delicate balance of secondary structure propensity in these N-terminal regions, which together play a key role in modulating fibrillation in opposing and complementary ways.

## Material and Methods

### αSyn expression and purification

#### NMR Samples

The expression and purification of all non-acetylated WT and variant αSyn were described previously [39]. Briefly, pelleted cells were suspended in PBS and homogenized three times at 10,000-15,000 psi. The cell lysates were centrifuged and streptomycin sulfate (Fisher) (10 mg/ml) was added to the supernatant and the mixture was stirred at 4 °C for 30 minutes, followed by centrifugation. Ammonium sulfate (Sigma) (0.361 g/ml) was added to the supernatant and the mixture was stirred at 4 °C for 30 minutes, followed by centrifugation. The pellet was re-suspended in PBS and boiled for 20 minutes. After the mixture cooled down, the mixture was centrifuged. The supernatant was dialyzed against 25 mM Tris HCl buffer overnight. The supernatants were then passed through a 0.22 µm filter before being loaded onto a Hitrap Q column on an AKTA FPLC system (GE Healthcare LifeSciences). The column was equilibrated with 25 mM Tris HCl pH 7.7, and acetylated αSyn was eluted by applying increasing concentrations of up to 500 mM NaCl. αSyn usually eluted at ~250 mM NaCl. The Syn enriched fractions were dialyzed into 20 mM ammonium bicarbonate, lyophilized, and stored at -20 °C. The expression and purification of acetylated WT and A53T were as previously described, using mild methods. Mild purification excludes the salting-out and boiling steps and utilized size exclusion chromatography as in a previous report. NMR samples are not sensitive to differences in the purification method. The monomer containing fractions after size exclusion chromatography were flash frozen and stored at -20°C instead of being lyophilized [20,40].

#### Fluorescence samples

Thioflavin T (ThT) fluorescence was repeated for WT, Ac-WT, A53T and Ac-A53T used in the four way comparison for this work. The samples were expressed and purified as the non-acetylated human-to-mouse chimera set except that the anion exchange fractions were flash frozen in liquid nitrogen and stored at -20°C.

### NMR chemical shift measurement and SSP analysis

Secondary structure propensities (SSPs) of eight human-mouse chimera αSyns are from a previous study, as is the SSP of Ac-WT αSyn. The SSP of Ac-A53T in this report is similarly obtained by using ^13^C^α^ and ^13^C^β^ chemical shifts as input and a five residue sliding window with Zhang et al. [41] random coil references. Chemical shifts for eight human-mouse chimeras, Ac-WT and Ac-A53T are deposited in the Biological Magnetic Resonance Bank (BMRB) (See Table S1). ^13^C assignments for Ac-A53T were obtained from a 350 µM ^15^N and ^13^C doubly labeled sample in 10% (v/v) D_2_O in PBS buffer at 15 °C on a Varian 800 MHz spectrometer. HNCACB/CBCACONH, HNCO/HNCACO and HNN triple resonance experiments were collected to obtain HN, ^15^N, CO, ^13^C^α^ and ^13^C^β^ resonance assignments. The color coded SSP boxplots are made by R software.

### ThT fluorescence

The fibril assembly kinetics data for eight human-mouse chimera αSyns are from a previous study. For Ac-WT, WT, A53T and Ac-A53T used in four-way comparison, all the proteins were expressed and purified at the same time using the purification methods described in the αSyn expression and purification section. The proteins were then buffer exchanged to PBS pH 7.4 buffer using 10 kD centrifugal filters. Before aliquot distribution on a 96 well plate, the protein solutions are filtered with 100 kD centrifugal filters. The final protein concentration was 150 µM with 20 µM ThT for fluorescence measurements. An amount of 100 µL of the mixture was then pipetted into a well of the 96-well clear-bottom black-wall plate from Costar and sealed with clear sealing film from Axygen to prevent the evaporation during incubation. Measurements were recorded at 37 °C with linear shaking at 600 rpm. ThT fluorescence was recorded at 30 minute intervals using a POLARstar Omega reader from BMG. At least six replicates were performed on each protein, and sigmoidal fitting were used to obtain lag time and *k*_*app*_. The average of replicates is taken as the mean and the standard deviation of replicates is taken as error. The fibril morphology of Ac-A53T was observed with negative staining TEM as previously described [39].

## Results

### Fibril growth rates in non-acetylated human-mouse chimeras are highly correlated with secondary structure propensity changes in the N-terminal residues 5–8, 14–31 and 50–57

In a previous report [39] we have shown that there is a qualitative relationship between enhanced transient helical secondary structure around residues 53 and fibril growth rates in a set of eight non-acetylated human-to-mouse αSyn chimera proteins that were designed to contain one, two, or three domains of human or mouse αSyn. The eight possible chimeras are named for sequence identity to human or mouse αSyn in the N, NAC or C-terminal regions (Figure 1A) and the mutations from human or mouse αSyn and naming are outlined in Table S1. They include the WT human αSyn (HHH) and the human A53T mutant (MHH), which we examine the effect of acetylation upon later in this report. Using these eight variants, we extend our previous observations of monomer transient secondary structure into a quantitative analysis to evaluate; 1) whether there is a quantitative correlation between secondary structure propensity of the monomer and the growth rates and lag phases for the eight human-mouse variants and 2) to establish the specific locations of secondary structure propensity within the sequence of αSyn that are correlated to growth rate (*k*_*app*_).

A boxplot representation of secondary structure propensity changes using the SSP index [42] as a function of residue across the eight variants is color coded to show which residues’ SSP value in the sequence have a strong positive correlation (red) to *k*_*app*_ (Figure 1B, BMRB accession numbers for the chemical shifts are indicated in Table S1). Strikingly, contiguous residues in three distinct regions of the N-terminus show a strong positive correlation (r>0.7) between increased SSP and fibril growth rates, corresponding to increased transient helix at residues 5–8, 14–31 and 50–57 (Figure 1B). Changes in SSP values arising from chemical shift differences across the variants in the NAC and C-terminal regions generally do not show strong correlations with fibril growth rates, suggesting the importance of the N-terminus in directing *k*_*app*_. A more direct visualization of the correlation can be seen by plotting growth rates versus average SSP values across the three identified regions (Figure 1D). In fact, this visualization, which accounts for the effect in all three regions instead of at each residue, only enhances the relationship (r=0.93) and suggests the cumulative and significant effect of transient helical structure in these three regions upon *k*_*app*_.

### Lag times in non-acetylated human-mouse chimeras are correlated with secondary structure propensity changes in the N-terminal 51–55 region and the NAC 84–87 region

Similarly to the quantitative analysis for growth rates, we also examined the quantitative correlation between lag times and secondary structure propensities for the eight human-mouse variants. Although the correlation is not as strong (r>0.5 & r<-0.5) as the correlation between secondary structure propensity and *k*_*app*_, lag times are known to be sensitive to many variables, and a correlation exists between lag times and secondary structure propensity for residues 51–55 and 84–87. The boxplot representation of SSP changes as a function of residue across the variants is color coded to show which residues of the sequence have a positive correlation (yellow) and negative correlation (green) to lag times (Figure 1C). The positive correlation in region 51-55 of the N-terminus indicates that increased SSP are correlated to lag times, and the negative correlation in region 84-87 of the NAC region indicates that decreased SSP (increased β-structure) are correlated to lag times. As mentioned, increased SSP is associated with increases in transient helical population, whereas more negative SSP values are correlated with increased β structures in these regions. When we combine the residue-specific effects observed in these two regions by taking the average SSP in region 51–55 minus the average SSP in region 84–87 and plot these percentages per variant versus lag times, a stronger correlation relationship arises within the set, with a correlation coefficient of r = 0.75 (Figure 1E). While fibril elongation rates are correlated only to the N-terminal regions of the protein, the fibril lag phases are correlated to both the N-terminus and NAC regions.

### N-terminal acetylation of the familial A53T mutation: role of region specific transient helices in the fibrillation sensitive N-terminal region

Acetylated-A53T (Ac-A53T) is of great interest to study as it is the more physiological form of the A53T early onset PD mutant. Ac-A53T is also interesting from a biophysical perspective, as this variant has the potential to possess both fibrillation-promoting transient secondary structure due to the A53T mutation and fibrillation inhibiting transient secondary structure arising from modification by the N-terminal acetyl group. In order to understand the relationship between the fibrillation promoting region (residues 5–8, 14–31 and 50–57) determined from the human-mouse chimeras, and the fibrillation inhibiting residues (residues 1–12) determined from N-terminal acetylation, we examine the Ac-A53T variant in this report. In previous studies we have shown that N-terminal acetylation of WT αSyn alters the SSP values in residues 1–12 and slows down fibril growth rates by approximately a factor of two. Ac-A53T is a link between the delayed growth rates that arise from N-terminal acetylation of WT αSyn, accelerated fibrillation arising from the A53T substitution, and their associated transient secondary structural propensities, reflected in SSP values. Chemical shifts for the acetylated mutant and WT are deposited into the BMRB; accession numbers are listed in Table S1.

**Figure 2:**
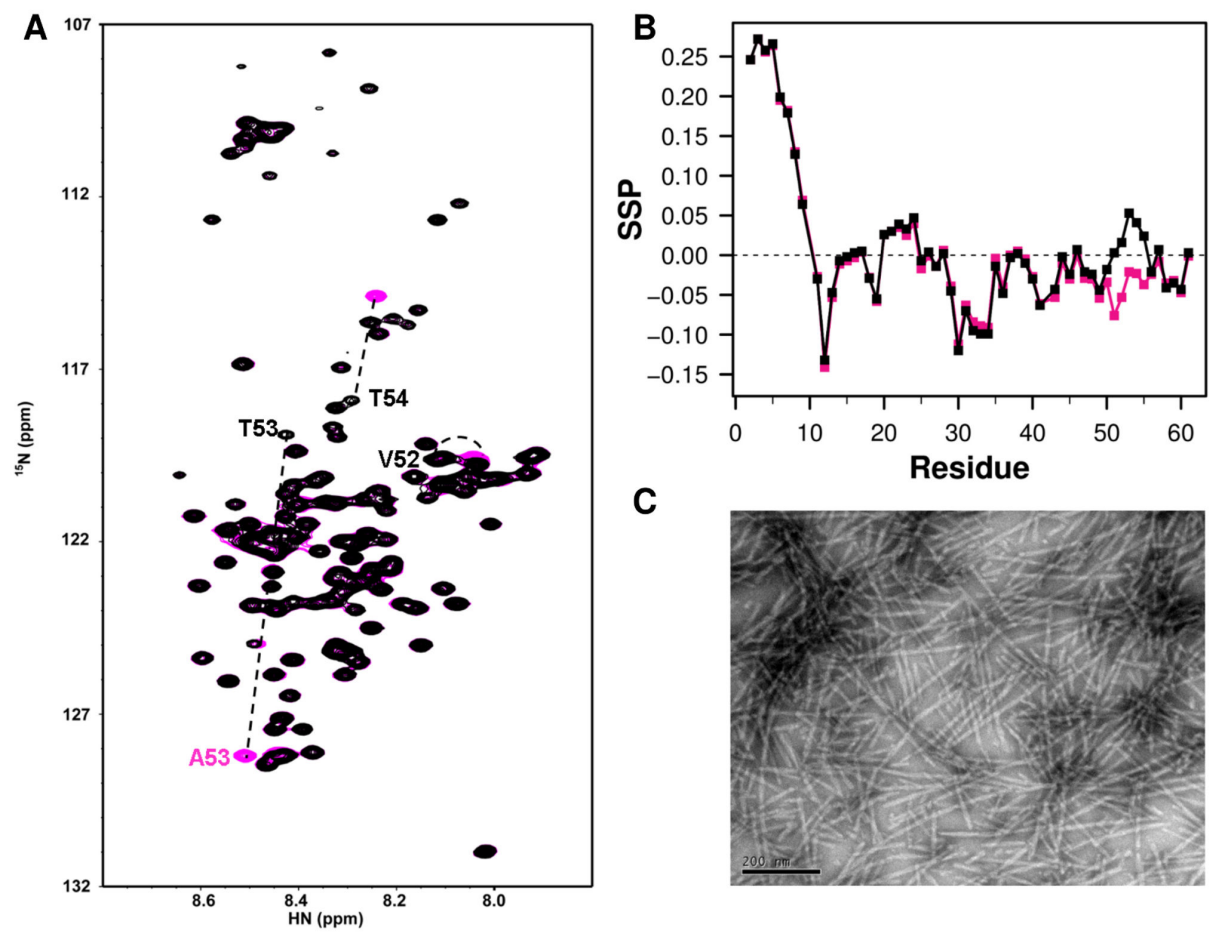
NMR and fibril morphology comparison of Ac-WT and Ac-A53T. A. Overlay of ^15^N-^1^H HSQC of Ac-A53T (black) and Ac-WT(magenta) at 15 °C in PBS buffer at pH 7.4. B. SSP analysis of Ac-WT (magenta) and Ac-A53T (black) plotted for the N-terminal region. The overlay of the rest of the proteins can be seen in Figure S2. C. Negatively stained electron micrographs of the end products of fibril formation of Ac-A53T fibril. The scale bar is 200 nm.

We draw together what we have learned from the correlations in the human-to-mouse chimera set and initial investigations of the acetylated WT in a four-way comparison of secondary structure propensities and fibril assembly kinetics of Ac-A53T, Ac-WT, A53T and WT αSyn. Previously, we and others have demonstrated increased N-terminal helix between the *in vitro* Ac-WT and WT monomers and similar fibril morphology of Ac-WT and WT fibrils, although Ac-WT fibrils form at a slower rate [20]. Similar comparisons can be made for A53T monomer and fibril relative to WT although A53T fibrillation kinetics are faster. The HSQCs of the non-acetylated WT and A53T mutant differ around position 53 as shown in Figure S2. In this report we compare the more physiological Ac-A53T against Ac-WT for the first time. Not surprisingly, the HSQC spectrum (Figure 2A) of Ac-A53T is very similar to the spectrum of Ac-WT except around the mutation site at residue 53 [20], which also corresponds to the biggest difference in SSP values (Figure 2B). This suggests that the N-terminal secondary structure propensity of Ac-A53T is similar to the Ac-WT αSyn conformation at the N-terminus, but that there are small differences in secondary structure propensity around the mutation site at A53T. The increased alpha helical propensity relative to the Ac-WT mirrors the trend seen in the non-acetylated chimera set for WT vs. A53T αSyn. The fibril morphology of Ac-A53T (Figure 2C) is indistinguishable from Ac-WT αSyn and shows the typical un-branched fibril morphology with a width of around 10 nm.

**Figure 3:**
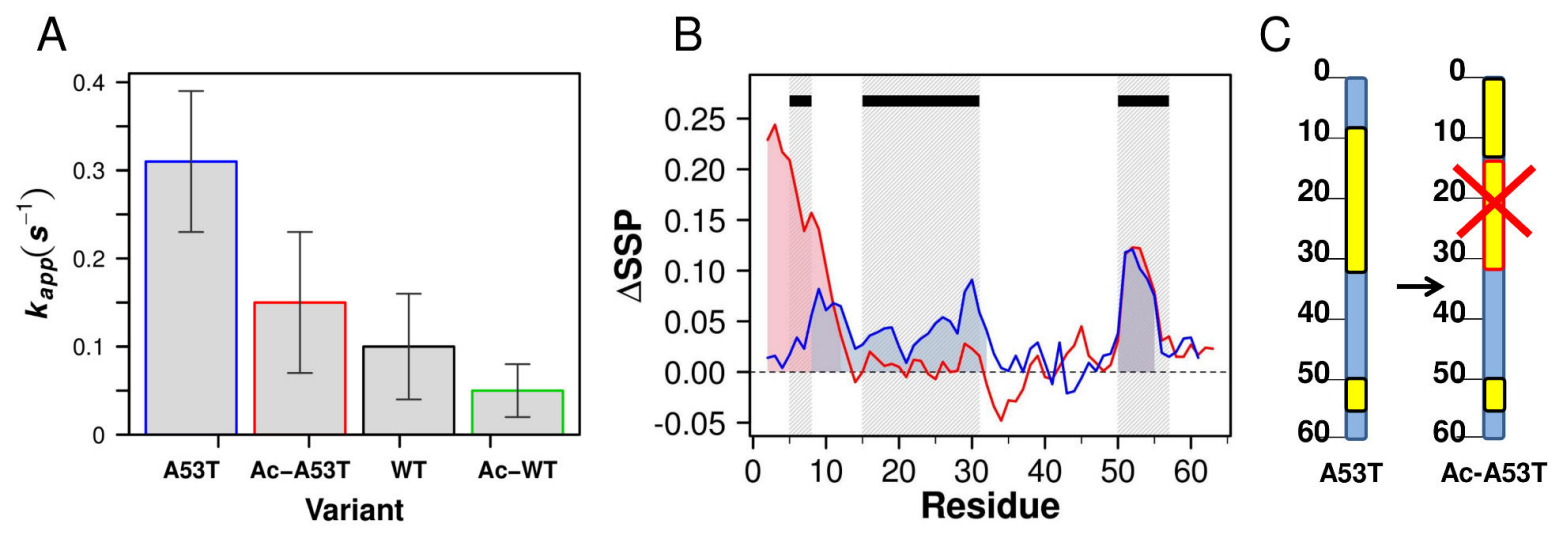
The effect of the A53T mutation upon the acetylated mutant. A. Histogram plot of the apparent rate of fibril growth, *k*_*app*_ of A53T (blue), Ac-A53T (red), WT (black) and Ac-WT (green) calculated by sigmoidal fitting of ThT fluorescence curves. Due to the different purification approach from the previously published one, the absolute *k*_*app*_ values are different, however the ratio relative to WT is the same. B. Differences of SSP shown for the N terminal residues 1 to 60 for Ac-A53T vs. WT (red) and A53T (blue) vs. WT; ΔSSP = SSP (Variant) -SSP (WT). The red and blue shading in the positive regions of the SSP curves correspond to increased transient helix observed for Ac-A53T and A53T relative the WT. The black rectangles at the top of the plot represent the regions of increased transient helix that are quantitatively correlated with accelerated growth rates from the chimera set (Figure 1B). The overlay of the shaded regions and the black rectangles highlights the overlapping boundaries between these data sets. These data in combination with the fibril kinetics suggest that increased helicity in residues 1-12 is fibril inhibiting, while increased helicity in residues14-31, 50-57 are fibril accelerating, and increased transient helix at 5-8 may depend on the overall context. C. A schematic representation of the secondary structure propensity differences in regions in A53T and Ac-A53T, based on the ∆SSP of Figure 3B. The blue rectangle represents the N-terminal sequence, the black scale the residue number, and the yellow blocks the increase of SSP values of the variants relative to WT. The red outline and an X in the block represents the removal, or inability to sample increased SSP in this region by N-terminal acetylation highlighting that transient helix supported by the A53T mutation in the non-acetylated protein is not supported alongside acetylation.

While all four proteins present similarly with respect to fibril morphology, their fibril kinetics point to fundamental differences between the four variants. The apparent fibril growth rates (Figure 3A) show clearly that the A53T mutant proteins fibrillate more quickly than the WT, and the acetylated proteins, Ac-A53T and Ac-WT αSyn, fibrillate more slowly than their non-acetylated counterparts with the order of fibrillation rate A53T> Ac-A53T> WT> Ac-WT αSyn. As the lag times of the four proteins have large standard deviations, we do not draw conclusions from analysis of the lag phases. We demonstrate that, similar to the chimera set, small but significant structural differences in the N-termini between these four proteins are the determinants of their respective fibrillation kinetics, as the NAC and C-terminal regions of all four proteins are otherwise quite similar by our analyses, as the SSP values generally overlay in these regions as shown in Figure S1.

Comparison of the secondary structure propensity of A53T and Ac-A53T relative to WT with an SSP difference plot highlights the ways these proteins differ from the non-acetylated WT (Figure 3B, 3C). In the non-acetylated A53T mutant, increased SSP is observed in both the local region near the site of mutation (residues 50–55) and in the non-local region (residues 8–32), indicating some long-range effects of the A to T amino acid substitution (Figure S2). In the acetylated protein, however, the impact of the A53T mutation is solely local and the effect of increased SSP in the region encompassing residues 8-32 is no longer observed in the mutant (Ac-A53T) (Figure 3C). The difference SSP plot of Ac-A53T versus WT αSyn also shows increased SSP at the most N-terminal portion of this sequence which has been previously attributed [20] to modification by the acetyl group at the initiating methionine. While we observe increased helix at 1-12 as corresponding to delayed growth rates in the acetylated proteins, we also showed that increased transient helix at 5-8 was correlated with increased fibril kinetics in the chimera set (Figure 1, black lines in Figure 3B). We emphasize that we have not quantitatively correlated the entire region 1-12 with inhibition, but the data implied here suggest that increased transient helix at 5-8 may function differently when in the context of acetylation versus the A53T mutation alone. We also observe a distinct difference in the magnitude of the transient helices. Effects of N-terminal acetylation are several times greater than effects from the simple A53T mutation, which may explain why transient helix at 1-12 may override effects of transient helix at 5-8 from the A53T mutation. The SSP profile of Ac-A53T is not the direct sum of the A53T mutation and N-terminal acetylation; rather, it is the sum of the local effects of N-terminal acetylation at residue 1 and the local effects of the A53T mutation (Figure 3C). It differs from the non-acetylated A53T in that the acetylated protein does not support non-local increased SSP of residues 8–32, of which we identify the region 14-31 as a key fibril growth accelerator based on the chimera studies (Figure 1B, 1D and 3B). This pattern of SSP values is consistent with the observed fibrillation kinetics. A schematic diagram highlighting the regions of secondary structure change represented in the ∆SSP plot shows these features clearly (Figure 3B, 3C). Increased N-terminal helix appears to have opposing effects on the kinetics of fibril formation depending on its magnitude and the specific region in which it occurs. We suggest that the decrease in fibril assembly kinetics due to N-terminal acetylation in the A53T mutant may arise from either or both of these structural reasons: 1) the formation of some portion of transient helix in residues 1–12 alone and/or 2) the disruption of “fibrillation promoting” transient helix at residues 14–31.

## Discussion

### Molecular description of αSyn fibril assembly: mechanistic implications for N-terminal acetylation

**Figure 4:**
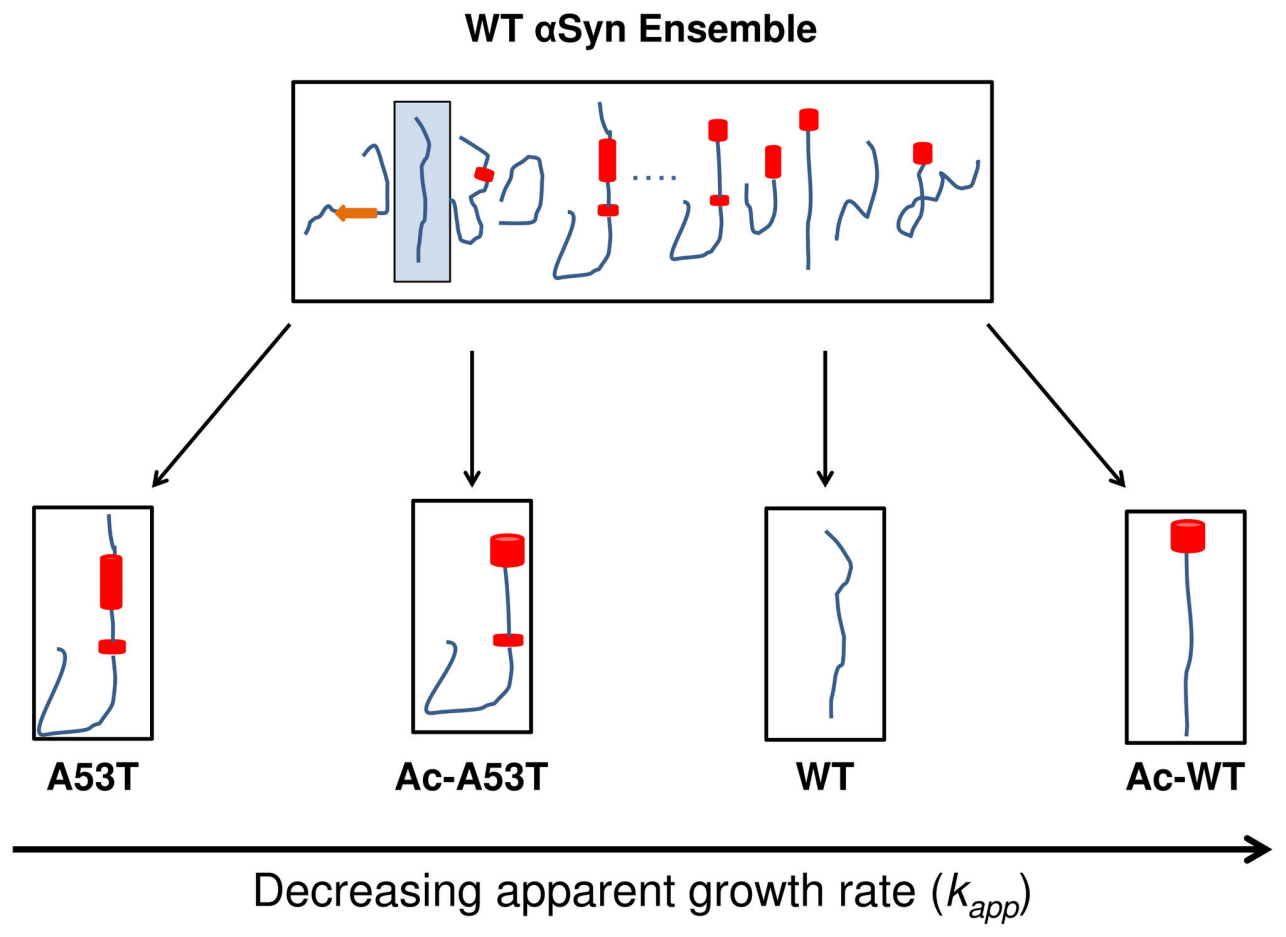
Schematic diagram of a conformational selection and population shift mechanism for A53T and acetylated proteins. The representation of the population distribution is based on a description by Ma et al. The populations of the various monomer species in the fibrillation process are represented by blue curves. Monomer conformations exist in a heterogeneous ensemble and are shown by schematic drawings with the red cylinder representing α-helix. The disordered monomers sample large heterogeneous ensembles that are differentially populated; both fibrillation prone and non-fibrillation prone monomers exist within the heterogeneous ensemble. Sequence modifications shift the population to favored conformations, which are boxed. The increase in transient helix propensity of the modified protein relative to the WT αSyn is presented. Secondary structure propensity correlations with fibril growth rates suggest that the fibrillation prone conformation consists of increased transient helix in residues 14–31 and 50–57 relative to WT, while transient helix in residues 1–12 arising from N-terminal acetylation is fibrillation inhibiting. By definition, the WT protein does not have fibrillation prone regions, and Ac-A53T which contains both aggregation promoting (residues 50–57) and fibrillation inhibiting conformations (residues 1–12) has similar fibrillation behavior to WT. Ac-WT, which contains only the fibrillation inhibiting conformation (residues 1–12) fibrillates the slowest.

We present a schematic view of a conformational selection model for αSyn that relates the secondary structure propensities in the N-terminus to the fibrillation rates based on the human-mouse chimera results and the data from Ac-A53T (Figure 4). The disordered WT αSyn monomer we observe is in actuality a large heterogeneous ensemble that is differentially populated with both fibrillation prone and non-fibrillation prone monomers. Sequence modifications, by N-terminal acetylation or mutation, shift the population distributions of the αSyn species toward different secondary structures and, therefore, different fibrillation kinetics. In other words, the A53T mutation increases the transient helical population in several N-terminal regions and N-terminal acetylation increases the population of transient helix at any given point in the region 1-12, and these population shifts are enough to dissuade or promote the protein to form fibril. This makes the fibrillation of Ac-A53T which only contains one region of accelerating helix (50-55) faster than Ac-WT αSyn that contains only the inhibitory transient helix 1–12, but slower than A53T that contains accelerating transient helix at both 14-31 and 50-55. These results highlight the importance of shifting future *in vitro* work to the acetylated protein. While the helix which forms non-locally from the A53T mutation in the non-acetylated A53T is correlated to acceleration, it occurs at a small magnitude, reflected in the smaller SSP values. The helix arising from acetylation is a fairly substantial portion of the population, and quite stable, even at high pressures [43], further corroborating alongside our work here, the relevance of its presence to the ensemble. Furthermore, these results highlight that transient helix is not broadly accelerating when it occurs in the N-terminus, but that it depends on the region in which it occurs.

The mechanism of αSyn fibril assembly from intrinsically disordered monomer ensemble to amyloid fibril is complex and includes a macroscopic lag and growth phase, during which the monomer form of αSyn undergoes a nucleation dependent conversion to a cross β-rich fibril form [44]. Here, we have examined the relationship between the monomer secondary structural propensity and fibril assembly kinetics. New molecular recognition paradigms for protein fibrillation include proposals for a conformational selection/population shift process [45,46] which originates in the monomer ensembles. We show an excellent correlation between the change in secondary structure propensity of the monomer with *k*_*app*_ and good correlation with lag times, suggesting that indeed the conformational features of the monomer species are critical to the mechanism of fibril formation consistent with a conformational selection model for fibril assembly. We also emphasize that we only consider fibrillation. For example, although the total aggregation kinetics of A30P familial mutant is faster, A30P has slower fibril assembly rates, (one subset of aggregation) than WT αSyn. Consistent with our picture of the faster fibrillating species, which shows increased helical propensity in the region around 30 relative to WT, A30P shows reduced helical propensity around residue 30 [47] and forms fibrils slower than WT.

Pre-existing conformational features, or fibrillation inhibited/prone monomer conformational ensembles, exist in solution prior to fibril assembly. The conformational sub-states of the monomer that are important in directing fibril formation in αSyn do not necessarily resemble the final fibril state but rather serve as fibrillation initiators or gatekeepers to the process. Not surprisingly then, many regions of αSyn are involved in the complex regulation of fibrillation, including regions that remain outside the fibril core once it forms. Although it is counter-intuitive to have non-fibril core regions affecting fibril assembly kinetics, it has been directly shown that the non-fibril core N-terminal helix plays a crucial role in initiating and accelerating fibrillation processes associated with Huntington’s disease and TTR1 [28,48,49,50]. Here, for αSyn, the transient helix sampled by the N terminus at residues 1–12 and 8-32 are not in the fibril core of the protein which has been determined by NMR to be from residues 30–110. However they are correlated, by implication or quantitative correlation respectively, to *k*_*app*_. This suggests that the monomer conformation in the non-core region is critical to monomer addition to the pre-febrile species. In contrast, residues 51-55 and 84-87 that are in the core of the fibril are associated with the lag phase. These results suggest that the monomer conformation in the fibril core region is important for the formation of the nucleus and the initiation of the fibril assembly process. Together the effects in these regions play part in this multi-step process. Here we present a complementary view that suggests that conformational features within the region of the fibril core are important in initiating fibril assembly and those in the non-core N-terminal region are critical to fibril elongation. Our observations correlating increased transient helix/β-structure to fibril kinetics represent the first steps in understanding the conformational switch αSyn experiences from disordered monomer to amyloid fibril.

## Conclusion

The results on the human-mouse chimera support the conclusion that secondary structure propensity of the monomer plays a critical role in directing fibril formation, and support the rationalization of fibril kinetics in the four-way comparison from WT to Ac-A53T. While we identify the N-terminal transient structure as playing the most critical role in the differences between these species, it is worth mentioning that N-terminal structure may not exist in isolation of long-range contacts, and long-range contacts involving or not involving the N-terminus may also critically direct monomer fibril assembly. Additionally, because N-terminal acetylation directly affects N-terminal transient structure and fibril kinetics in both the WT and A53T mutant, it is a relevant physiological modification to *in vitro* work. We proposed that the N-terminal helix arising from acetylation may inhibit the formation of fibrillation prone transient secondary structure that has been shown to be key fibril growth accelerators based on human-chimera variants. The acetylated A53T is an example of a variant that contains distinct regions of transient secondary structure within the N-terminus that are fibrillation inhibiting and fibrillation promoting. These regions offset one another and together ultimately determine the rate of fibril growth. Taken together, these data provide the basis for a mechanistic view of the role of acetylation in αSyn and suggest that this modification plays a role in inhibiting fibrillation. Further investigation of the nature of the mechanism of inhibition may be useful in designing anti-amyloidogenic molecules that target the monomer conformational ensemble.

## Supporting Information

Figure S1 **SSP overlay of Ac-WT, Ac-A53T, WT, and A53T.** The SSP values for the full-length A53T (blue), Ac-A53T (red), WT (black) and Ac-WT (green) are displayed per residue.
(TIFF)

Figure S2 **^15^N HSQC of the non-acetylated A53T and WT.** The A53T mutant is indicated by blue, and the WT by black. The few places they do not overlay are near the site of the A53T mutation.
(TIFF)

Table S1 **Description of variant naming and corresponding BMRB accession numbers.**
(DOCX)
