# Supplementary figures and images for: Mechanistic Insight into the Relationship between N-Terminal Acetylation of α-Synuclein and Fibril Formation Rates by NMR and Fluorescence
Source: PLoS One. 2013 Sep 18;8(9):e75018. doi: 10.1371/journal.pone.0075018 (PMC3776725; doi:10.1371/journal.pone.0075018)

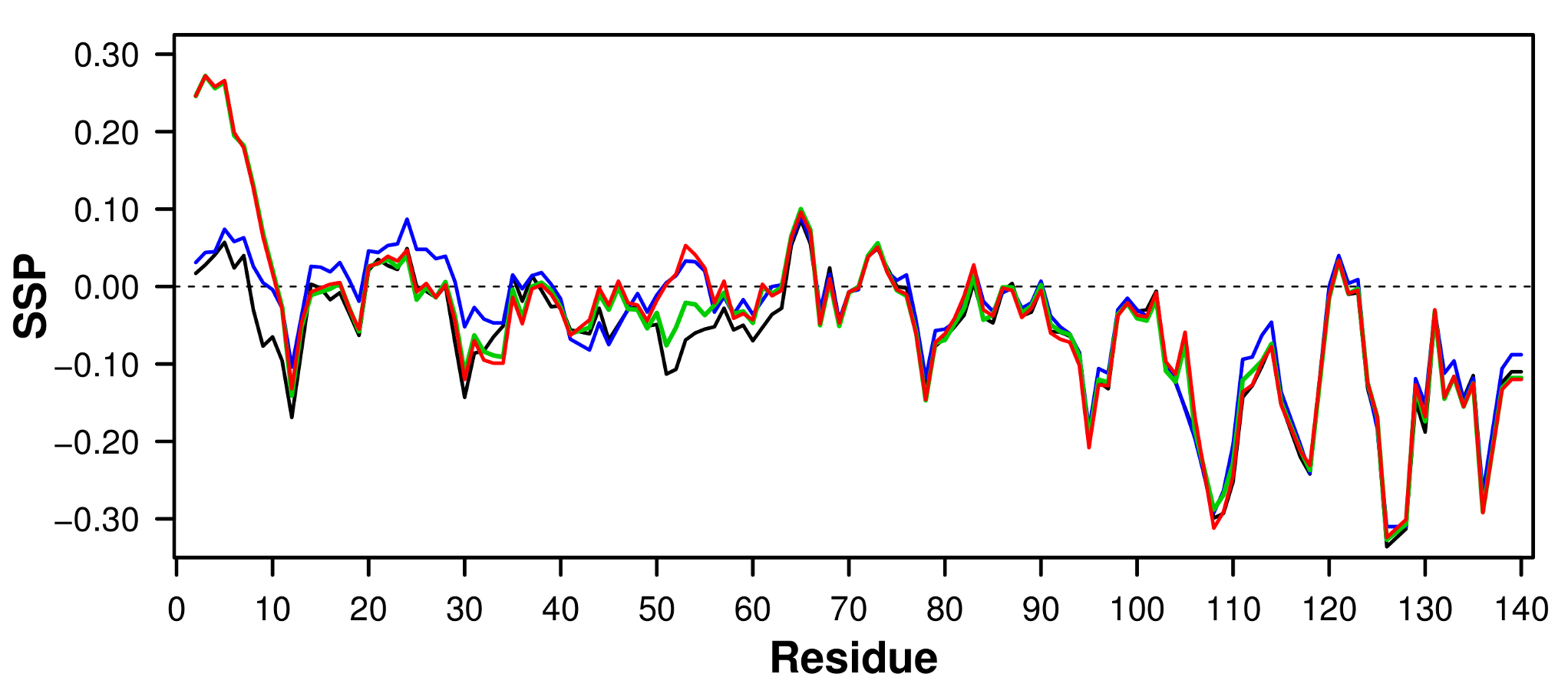

Supplement: Figure S1 — SSP overlay of Ac-WT, Ac-A53T, WT, and A53T. The SSP values for the full-length A53T (blue), Ac-A53T (red), WT (black) and Ac-WT (green) are displayed per residue. (TIFF) [file pone.0075018.s001.tiff]

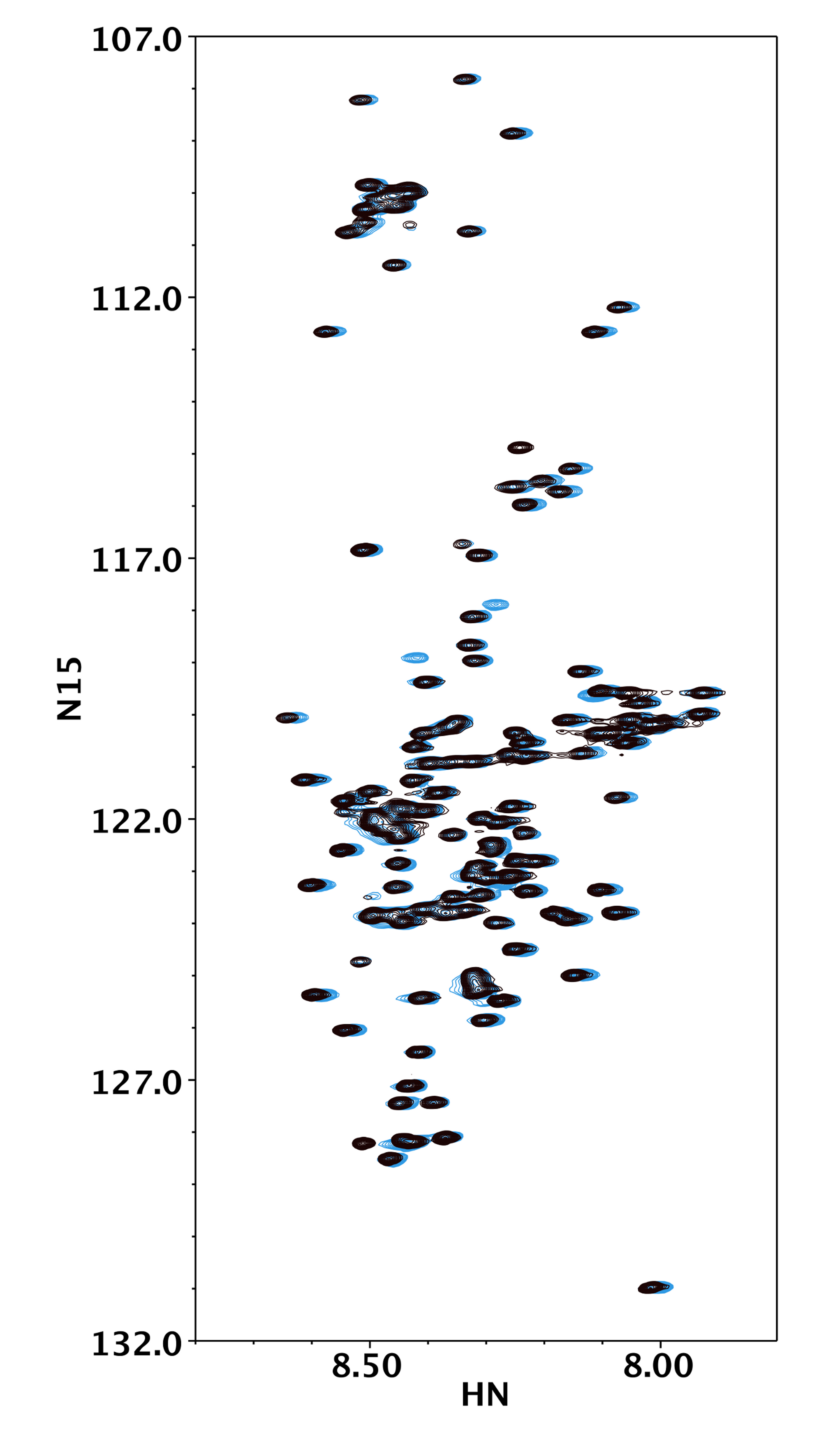

Supplement: Figure S2 — 15N HSQC of the non-acetylated A53T and WT. The A53T mutant is indicated by blue, and the WT by black. The few places they do not overlay are near the site of the A53T mutation. (TIFF) [file pone.0075018.s002.tiff]
