# Supplementary material for: Mechanistic Insight into the Relationship between N-Terminal Acetylation of α-Synuclein and Fibril Formation Rates by NMR and Fluorescence
Source: PLoS One. 2013 Sep 18;8(9):e75018. doi: 10.1371/journal.pone.0075018 (PMC3776725; doi:10.1371/journal.pone.0075018)
Supplement: Table S1 — Description of variant naming and corresponding BMRB accession numbers. (DOCX) [file pone.0075018.s003.docx]

**Table S1. Description of variant naming and corresponding BMRB accession numbers.**

| Human-Mouse αSyn chimera set[[1](#_ENREF_1)]. | This Work. | Description. | BMRB Accession Number. |
| --- | --- | --- | --- |
| --- | Ac-WT | Acetylated h-αSyn | 19350 |
| --- | Ac-A53T | Acetylated h-A53T-αSyn | 19351 |
| HHH | WT | Non-acetylated h-αSyn | 19337 |
| MHH | A53T | Non-acetylated h-A53T-αSyn | 19338 |
| HMH | --- | Non-acetylated aSyn h-S87N | 19344 |
| MMH | --- | Non-acetylated aSyn h-A53T & h-S87N αSyn | 19345 |
| MMM | --- | Non-acetylated m-αSyn | 19346 |
| HMM | --- | Non-acetylated aSyn m-T53A-αSyn | 19347 |
| MHM | --- | Non-acetylated aSyn m-N87S-αSyn | 19348 |
| HHM | --- | Non-acetylated aSyn m-T53A & m-N87S αSyn | 19349 |

**References**

1. Kang L, Wu K-P, Vendruscolo M, Baum J (2011) The A53T mutation is key in defining the differences in the aggregation kinetics of human and mouse α-synuclein. Journal of the American Chemical Society 133: 13465-13470.
